# Hyaluronic acid is a negative regulator of mucosal fibroblast-mediated enhancement of HIV infection

**DOI:** 10.1038/s41385-021-00409-3

**Published:** 2021-05-11

**Authors:** Johanne H. Egedal, Guorui Xie, Thomas A. Packard, Anders Laustsen, Jason Neidleman, Konstantinos Georgiou, Satish K. Pillai, Warner C. Greene, Martin R. Jakobsen, Nadia R. Roan

**Affiliations:** 1 Department of Biomedicine, Aarhus University, Aarhus, Denmark; 2 Gladstone Institute of Virology, Gladstone Institutes, San Francisco, CA USA; 3 Department of Urology, University of California, San Francisco, CA USA; 4 Vitalant Research Institute, San Francisco, CA USA; 5 Department of Laboratory Medicine, University of California, San Francisco, CA USA; 6 Departments of Medicine and Microbiology & Immunology, University of California, San Francisco, CA USA

## Abstract

The majority of HIV infections are established through the genital or rectal mucosa. Fibroblasts are abundant in these tissues, and although not susceptible to infection, can potently enhance HIV infection of CD4+ T cells. Hyaluronic acid (HA) is a major component of the extracellular matrix of fibroblasts, and its levels are influenced by the inflammatory state of the tissue. Since inflammation is known to facilitate HIV sexual transmission, we investigated the role of HA in genital mucosal fibroblast-mediated enhancement of HIV infection. Depletion of HA by CRISPR-Cas9 in primary foreskin fibroblasts augmented the ability of the fibroblasts to increase HIV infection of CD4+ T cells. This amplified enhancement required direct contact between the fibroblasts and CD4+ T cells, and could be attributed to both increased rates of *trans*-infection and the increased ability of HA-deficient fibroblasts to push CD4+ T cells into a state of higher permissivity to infection. This HIV-permissive state was characterized by differential expression of genes associated with regulation of cell metabolism and death. Our results suggest that conditions resulting in diminished cell-surface HA on fibroblasts, such as genital inflammation, can promote HIV transmission by conditioning CD4+ T cells toward a state more vulnerable to infection by HIV.

## Introduction

The primary route for HIV transmission is through the genital and rectal mucosa following sexual intercourse.^1^ The most abundant cells within mucosal tissues are epithelial cells (ECs) and stromal fibroblasts. ECs, which line the lumen of mucosal tissues, are the first cells to encounter HIV during mucosal transmission. Under circumstances where the epithelium is breached (e.g., as a result of traumatic infectious ulcerations),^2^ HIV can access the underlying stromal compartment wherein fibroblasts are abundant. We recently demonstrated that although mucosal stromal fibroblasts are not susceptible to productive infection by HIV, they potently enhance HIV infection of CD4+ T cells.^2^ The mechanisms behind this enhancement are not fully elucidated, but seem to be mediated through a combination of *trans*-infection of CD4+ T cells by fibroblasts (whereby fibroblasts can capture HIV virions on their surface without becoming productively infected and transfer the virus to T cells^2^) and the fibroblasts’ ability to condition CD4+ T cells to become more permissive to infection.

Fibroblasts are not only important structural scaffolds of tissues, but also play essential roles in tissue homeostasis, tissue damage repair, and wound healing.^3^ They produce and are encapsulated by an extensive, biologically active extracellular matrix (ECM), of which hyaluronic acid (HA) is a major component. HA is a non-sulfated glycosaminoglycan (GAG) composed of repeating β(1–4)-glucuronic acid and β(1-3)-N-acetyl glucosamine heterodimers.^4–6^ HA is predominantly produced by fibroblasts by three different synthases: HAS1, HAS2, and HAS3, all of which span the plasma membrane and release HA onto the cell surface and into the ECM.^5,6^ HA exists in two forms—high- and low-molecular weight HA (HMW HA/LMW HA). HMW HA is the principal HA product and is predominantly produced by HAS2. LMW HA is primarily a product of degraded HMW HA that can be generated by the enzyme hyaluronidase (HYAL), but can also be produced *de novo* by HAS3 (Fig S1a).^7^ Besides being a filler in connective tissue, HA is crucial for tissue hydration and the lubrication of joints and has important roles in embryogenesis, cell motility, signaling, and proliferation. Importantly, HA has also been reported to play important roles in responding to and regulating tissue inflammation. HMW HA is generally found under anti-inflammatory and immunosuppressive conditions, and upon acute inflammation is degraded to LMW HA. In contrast, LMW HA can induce the expression of pro-inflammatory genes and directly propagate inflammatory conditions.^4–9^ In the context of HIV, the role of HA is ambiguous. Exogenous HA (both LMW and HMW) has been reported to reduce HIV infection of CD4+ T cells, while enzymatic reduction of endogenous HA on the surface of CD4+ T cells increases their susceptibility to infection.^10^ On the other hand, endogenous HA on fibroblastic reticular cells (FRC) can help these cells *trans*-infect CD4+ T cells in secondary lymphoid organs.^11^ Therefore, whether HA helps or hinders HIV infection may be context-dependent. To what extent HA affects HIV infection in the context of mucosal tissues, the most common site of initial transmission, is unknown.

Because mucosal inflammation both increases susceptibility to HIV transmission and affects HA synthesis and catabolism, we investigated to what extent HA on mucosal fibroblasts influences fibroblast-mediated enhancement of HIV infection. We show that diminishing HA on fibroblasts, which mimic inflammatory conditions, increases the ability of the fibroblasts to increase HIV infection of CD4+ T cells, and further investigate the mechanisms underlying this phenomenon.

## Results

### Ablation of HAS2 in primary foreskin fibroblasts increases fibroblast-mediated enhancement of CD4+ T-cell infection by HIV

**Fig. 1:**
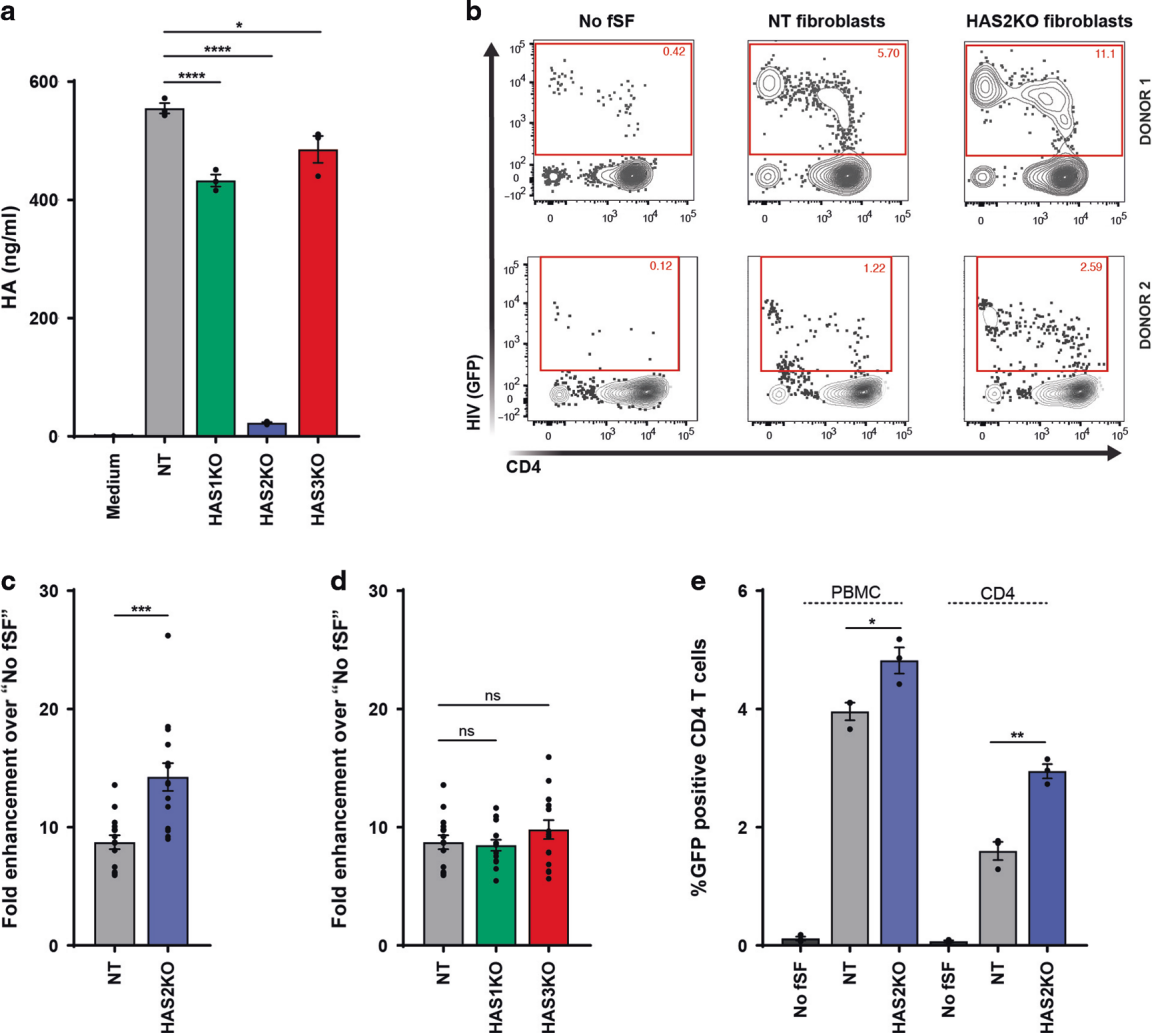
Ablation of HAS2 from primary foreskin fibroblasts improves their ability to enhance HIV infection of CD4+ T cells. **a** HAS2KO foreskin fibroblasts (fSFs) produce less hyaluronic acid (HA) than HAS2-sufficient fSFs. ELISA measuring HA concentration in the supernatants of fSFs treated with CRISPR guides against HAS1, HAS2, or HAS3, or a non-targeting (NT) guide as a negative control, following 24 h of culture in serum-free media. Data represent results from experimental triplicates. **b** HAS2KO fSFs increase HIV infection of CD4+ T cells more effectively than NT fSFs. Anti-CD3/CD28-activated PBMCs were infected with the reporter virus HIV^GFP^, either alone or in the presence of NT or HAS2KO fSFs. Percentages of productively infected cells, indicated within the gates, were measured 3 days post infection by flow cytometry. Shown are contour plots pre-gated on live, singlet CD3+ CD8− cells. Infected cells express low levels of CD4 due to downregulation of cell-surface CD4 by HIV.^64^ Each row corresponds to a different PBMC donor. **c** HAS2KO fSFs significantly increase HIV infection of CD4+ T cells. Bar graph showing infection rates in the absence or presence of the indicated fSFs, reported as fold-enhancement relative to infection in the absence of any fSF (NT: mean 8.72, SEM 0.59; HAS2KO: 14.24, SEM 1.18). Results are compiled from five donors each tested in triplicates. Infection rates were quantitated by flow cytometry as in (**b**). **d** HAS1KO and HAS3KO fSFs enhance HIV infection of CD4+ T cells at a similar rate to NT fSFs. Results are compiled from five donors each tested in triplicates. **e** Immune cells other than CD4+ T cells are not required for fSF-mediated enhancement of HIV infection. Bar graph showing infection rates in the absence or presence of the indicated fSFs, reported as percentage of infected cells, when infection was conducted with bulk PBMCs (“PBMC”) or with purified CD4+ T cells (“CD4”). Data correspond to results from experimental triplicates from one PBMC donor, and are representative of results from a total of four different PBMC donors. Error bars show mean with SEM. *P < 0.05, **P < 0.01, ***P < 0.001 as determined by an unpaired Student’s *t* test.

To investigate the effect of HA on fibroblast-mediated enhancement of HIV infection we used CRISPR/Cas9 editing to disrupt each of the three *HAS* genes individually in primary human foreskin stromal fibroblasts (fSFs) (Fig. 1). Gene disruption was validated by Sanger sequencing using Synthego’s Inference of CRISPR Edits (ICE) analysis (Fig S1b). As a functional readout for loss of HA, we implemented an ELISA to measure total HA levels in supernatants of the fibroblast knockout (KO) lines. The HAS2KO line produced minimal levels of HA (mean 22.7, SEM 1.5 ng/ml) relative to a control cell line that had been edited with a non-targeting (NT) guide (mean 555.0, SEM 8.7 ng/ml) (Fig. 1a). The HAS1KO and HAS3KO lines only exhibited small reductions in their production of HA relative to the NT line, despite high INDEL frequency (Fig. 1a and S1b). This was not unexpected as HAS2 has been shown to be the synthase producing most of the HA secreted from cells.^9^

We next investigated to what extent fSFs deficient in HA production affect HIV infection rates. PBMCs from uninfected donors were activated with anti-CD3/CD28 to render them permissive to HIV infection^12^ and then infected with an R5-tropic GFP reporter virus (HIV^GFP^)^2^ in the absence or presence of HAS2KO fSFs or the NT fSF control. Three days post infection, PBMCs were analyzed for HIV infection rates by flow cytometry (Fig S2). Consistent with published studies,^2^ the frequencies of infected CD4+ T cells increased significantly upon co-culture with NT fSF (from 0.4% to 5.7%). Interestingly, when cells were infected in the presence of HAS2KO fSFs, infection rates increased even further, up to 11.1% (Fig. 1b), a finding that was consistent among five out of five PBMC donors (Fig. 1c). HAS1KO and HAS3KO fSFs did not increase infection rates relative to NT fSFs (Fig. 1d), in line with these cells harboring similar levels of HA as the NT fSFs. HAS2KO fSFs also augmented the infection rate of purified CD4+ T cells (Fig. 1e). Overall, these results demonstrate that endogenous HA produced by HAS2 in fSFs limits the fibroblasts’ ability to enhance infection of CD4+ T cells by HIV.

### Exogenous HA does not inhibit fibroblast-mediated enhancement of HIV infection

**Fig. 2:**
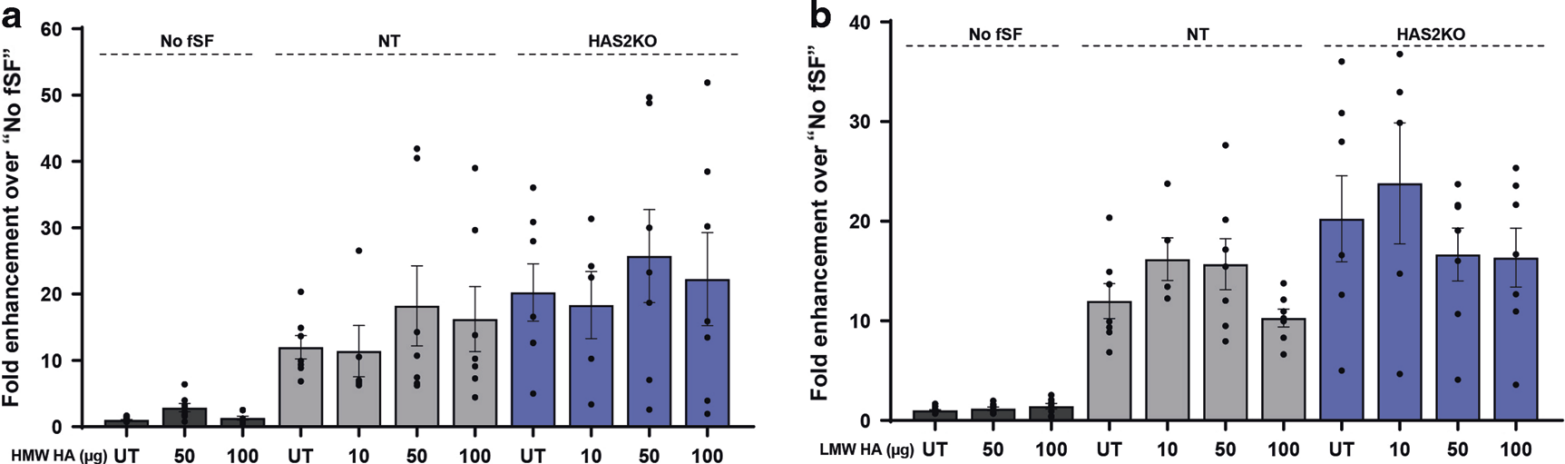
Exogenous HA does not diminish the infection-enhancing activity of HAS2KO fSFs. Anti-CD3/CD28-activated PBMCs were infected with HIV^GFP^, either alone or in the presence of NT or HAS2KO fSF. Prior to infection, HMW (**a**) or LMW (**b**) HA was added to 200 µl of the culture media at the levels indicated. Three days post infection, the frequency of GFP+ CD3+CD8− T cells was measured by flow cytometry. Shown are cumulative data from three experiments testing a total of five different PBMC donors. Results are reported as fold-enhancement of infection rates relative to HIV infection in the absence of fSFs. Error bars show mean with SEM. *P < 0.05, **P < 0.01, as determined by an unpaired Student’s *t* test.

It has previously been demonstrated that exogenous HA reduces HIV infection of unstimulated CD4+ T cells.^10^ Therefore, HAS2KO fSFs may enhance HIV infection more effectively than NT fSFs because they release less secreted HA. To investigate this possibility, we added exogenous HA to HAS2KO fSFs at levels matching those produced by NT fSFs (Fig. 1a). Neither LMW (15-40 kDa, Fig. 2a) nor HMW (>950 kDa, Fig. 2b) diminished the rate of HIV infection mediated by HAS2KO fSFs, suggesting that the infection-enhancing activity of NT fSFs is not simply dampened by the higher amount of HA they secrete.

### Superior infection-enhancing activity of HAS2KO fSFs requires direct contact with CD4+ T cells

**Fig. 3:**
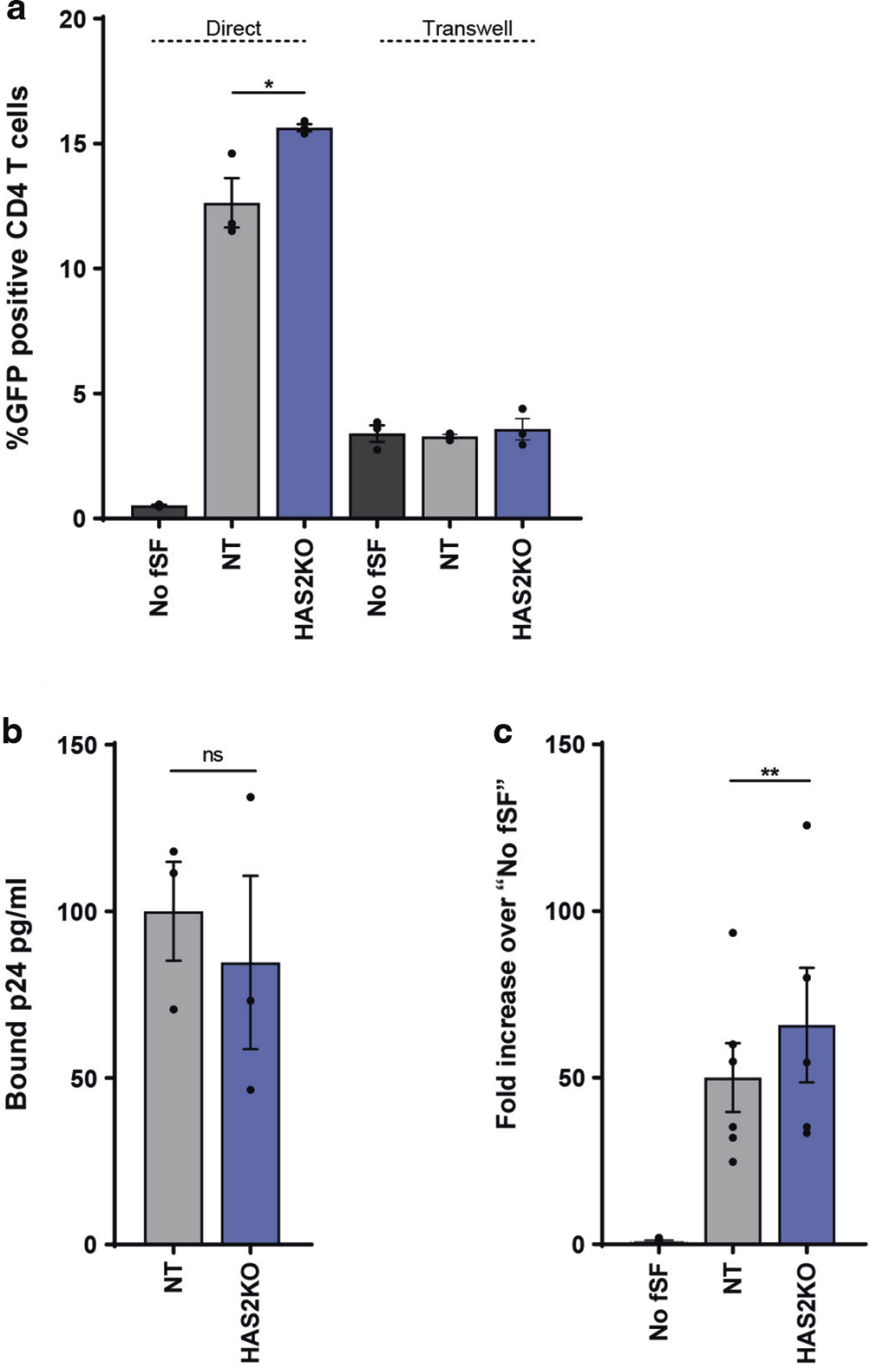
Superior infection-enhancing activity of HAS2KO fSFs is associated with increased *trans*-infection. **a** The superior infection-enhancing activity of HAS2KO over NT fSFs requires direct contact with CD4+ T cells. Activated PBMCs were infected with HIV^GFP^ in the presence of NT or HAS2KO fSFs. The PBMCs and fSFs were in direct contact during co-culture (“Direct”), or separated by a 1.0 µm pore-size transwell. Levels of productive infection were monitored 3 days post infection by flow cytometry. Data represent results conducted in experimental triplicates with one PBMC donor, and are representative of results from a total of three different PBMC donors. **b** HAS2KO fSFs capture similar amounts of HIV virions as NT fSFs do. NT and HAS2KO fSFs were incubated with HIV^GFP^ for 3 h at 37 °C. The amount of HIV particles bound to the fSFs was then measured by p24 ELISA following washing and cell lysis. Data represent results in experimental triplicates with one PBMC donor, and are representative of results from a total of two different PBMC donors. **c** HAS2KO fSFs *trans*-infect CD4+ T cells more efficiently than do NT fSFs. NT or HAS2KO fSFs were exposed to HIV^GFP^ for 2 h at 37 °C, and then washed to remove unbound virions. Activated PBMCs were added and infection rates were monitored 3 days later by flow cytometry. Wells lacking fibroblasts and pulsed with HIV^GFP^ were used as control. Data are compiled from two independent experiments each performed in triplicates. Error bars show mean with SEM. *P < 0.05, n.s. not significant, as determined by an unpaired Student’s *t* test.

We next investigated the possibility that other soluble factors secreted by the HAS2KO fibroblasts may increase HIV infection of CD4+ T cells. Activated PBMCs were added either directly to the NT or HAS2KO fSFs, or in a transwell system where the PBMCs were physically separated from the fSF. Subsequently, the cells were infected with HIV^GFP^. In the absence of fibroblasts, the transwell system resulted in higher infection rates than our regular culture conditions where transwells were not used, likely because of increased cell-to-cell contact between CD4+ T cells in the transwells. Neither NT nor HAS2KO fSFs further increased the rate of infection in the transwell system (Fig. 3a), demonstrating that the viral-enhancing activity of the HAS2KO line requires direct cell-to-cell contact with the CD4+ T cells.

As cell-to-cell contact is a hallmark of *trans*-infection, we next explored the possibility that the extent of HA “coating” on the fibroblast surface may dampen the ability of HIV to *trans*-infect from fibroblasts to CD4+ T cells. *Trans*-infection entails first the capture of HIV virions by fibroblasts, and then their presentation to CD4+ T cells. To test viral capture, NT and HAS2KO fSFs were incubated with HIV^GFP^ for 3 h. After extensive washing, the amount of virus bound to the cells was quantified by p24 ELISA. No significant differences in virus capture were observed between the two cell types (Fig. 3b). To test *trans*-infection more directly, the NT and HAS2KO fSFs were exposed to HIV^GFP^ for 2 h and then washed extensively before co-culture with activated PBMCs for 3 days. Surprisingly, even though the HAS2KO fSF did not capture more HIV^GFP^ than NT fSF (Fig. 3b), they transferred infectious virions to CD4+ T cells more efficiently than did NT fSFs (Fig. 3c). These results suggest that the superior infection enhancement by HAS2KO fSFs may be mediated in part by superior *trans*-infection even though the actual capture of virus by these cells is not more efficient.

### HAS2KO fSF condition CD4+ T cells to be more permissive to HIV infection

**Fig. 4:**
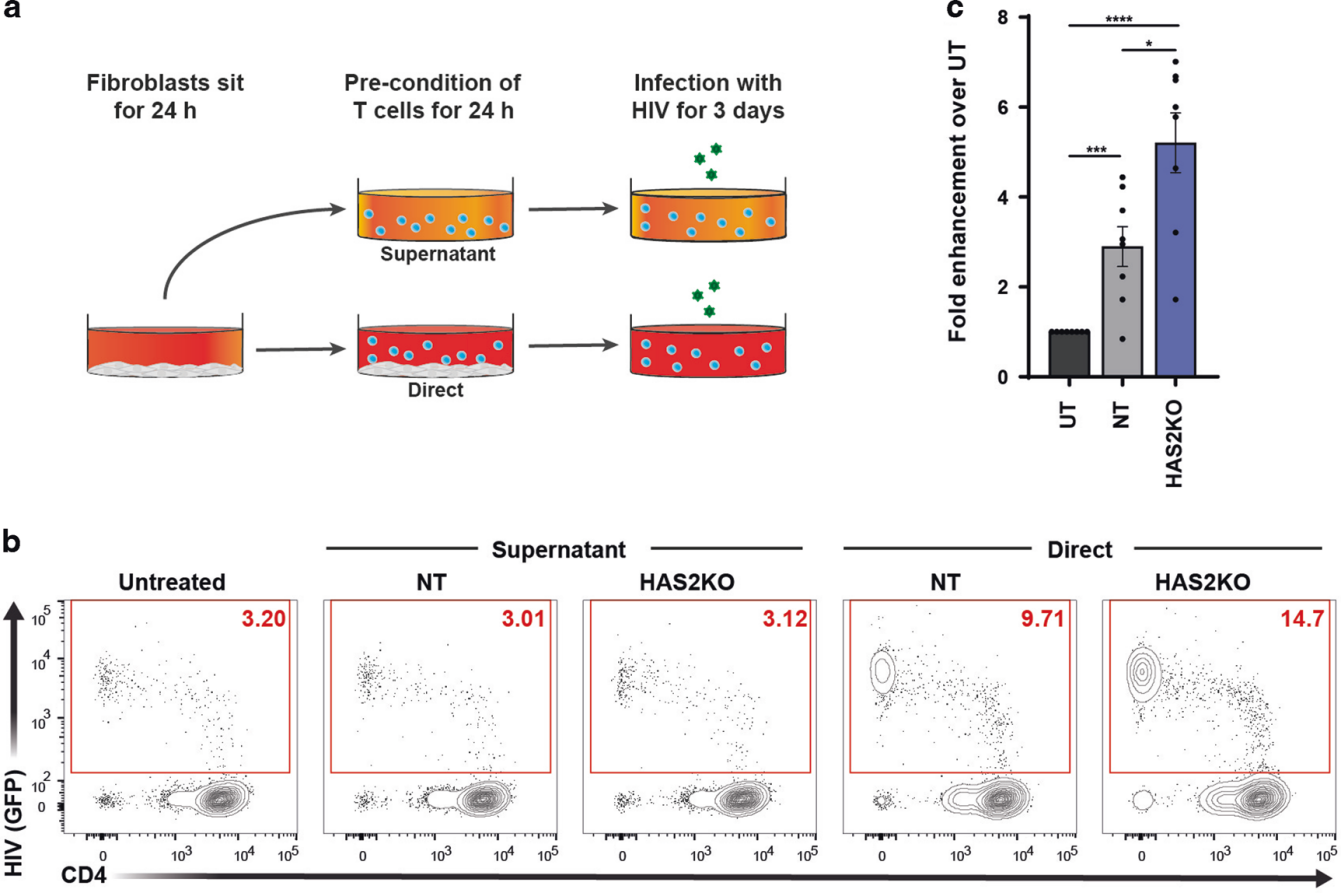
HAS2KO fSFs condition CD4+ T cells for HIV infection better than do NT fSFs. **a** Schematic of conditioning of CD4+ T cells by fSFs. Confluent fSFs were cultured for 24 h, after which activated CD4+ T cells were added either to the fSF supernatant or to the fSFs in fresh medium. Conditioning of the CD4+ T cells was allowed to proceed for 24 h before the cells were collected and infected with HIV^GFP^. Infection rates were assessed 3 days later by flow cytometry. **b** HAS2KO fSFs are more efficient than NT fSFs in conditioning CD4+ T cells to be more permissive to HIV^GFP^ infection. The experimental setup described in (**a**) was implemented to assess the extent of conditioning by HAS2KO vs. NT fSFs. Representative flow plots are shown. Numbers correspond to the percent of infected cells under the indicated treatment conditions. **c** HAS2KO fSFs are significantly more efficient than NT fSFs in conditioning CD4+ T cells. Conditioned CD4+ T cells were infected with HIV^GFP^ and assessed for infection rates 3 days later by flow cytometry. Data are reported as fold-enhancement relative to CD4+ T cells left untreated (UT) (NT: mean 2.90, SEM 0.44; HAS2KO: mean 5.21, SEM 0.67). Data represent results consolidated from eight different PBMC donors. Error bars show mean with SEM. *P < 0.05, **P < 0.01, ***P <  0.001 as determined by unpaired Students *t* test.

*Trans*-infection represents one mechanism of cell contact-dependent enhancement of CD4+ T cell infection, but is not the only one. We next investigated whether direct contact between HAS2KO fSF and CD4+ T cells can condition the latter to enter a state more permissive for HIV infection. CD4+ T cells isolated from activated PBMCs were pre-conditioned for 24 h by direct co-culture with NT or HAS2KO fSF, and then removed from the culture (Fig. 4a). As a control, CD4+ T cells were conditioned for 24 h with culture supernatants from the two types of fSFs. Conditioned CD4+ T cells were then exposed to HIV^GFP^, and assessed 3 days later for infection rates. Pre-conditioning with supernatant from NT or HAS2KO fSFs did not increase the infection rates of the CD4+ T cells (Fig. 4b), consistent with soluble factors not being important for fibroblast-mediated enhancement of HIV infection. In contrast, pre-conditioning with NT fSFs increased the infection rate up to fourfold, and with HAS2KO fSFs up to sevenfold (Fig. 4c). These results are consistent with the ability of fSFs to render CD4+ T cells more permissive for HIV infection,^2^ and suggest that the HA “coat” at the surface of fSFs normally dampens their ability to condition CD4+ T cells for infection.

### Conditioning by fibroblasts induces potent transcriptional changes in CD4+ T cells

**Fig. 5:**
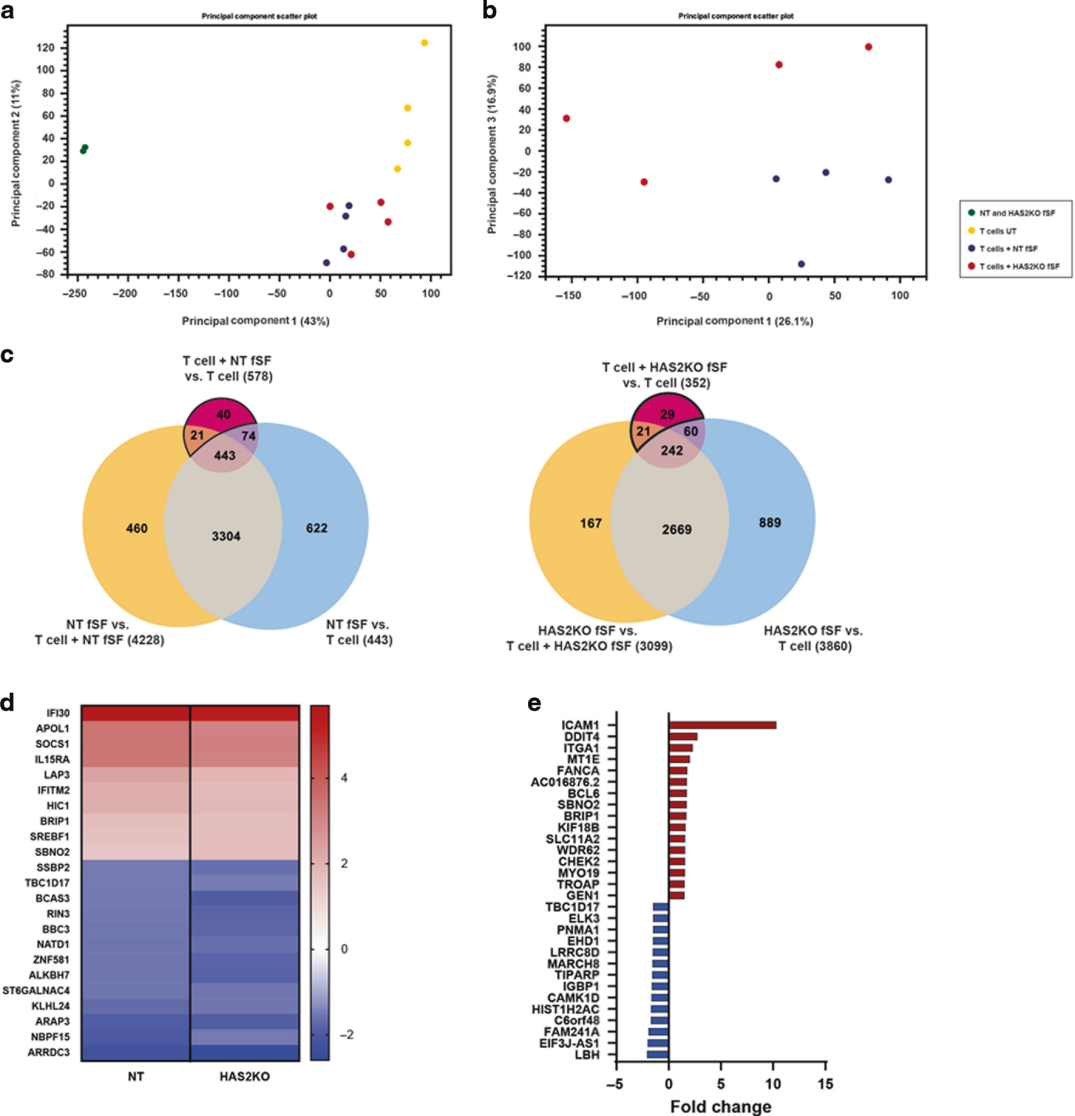
Conditioning of CD4+ T cells by NT and HAS2KO fSFs elicits transcriptional changes. **a**, **b** Principal component analysis (PCA) plot showing the clustering of fSFs alone (green), untreated CD4+ T cells alone (yellow) and CD4+ T cells conditioned by NT (blue) or HAS2KO (purple) fSFs (**a**), or the clustering of CD4+ T cells conditioned by NT fSFs relative to those conditioned by HAS2KO fSFs (**b**). **c** To exclude contaminating fibroblast genes from our differentially expressed gene (DEG) lists, we created a list of DEGs comparing the following three groups: T cells alone, fSFs alone, and T cells cultured with fSFs. Separate analyses were conducted for the NT and HAS2KO fSF cultures. To identify fSF-specific genes, the DEGs were compared in a Venn diagram (NT: left, and HAS2KO: right). Genes differentially expressed between non-conditioned and conditioned T cells are represented by the pink circles. These DEGs, however, can theoretically also include transcripts from contaminating fSFs. The blue circles represent DEGs between T cells and fSFs and correspond to transcripts unique to fibroblasts. The yellow circles represent DEGs between fSFs and conditioned T cells, and correspond to T cell-specific transcripts. To identify DEGs devoid of potential fSF-specific genes, we identified the DEGs in the pink circles and that did not overlap with those in the blue circles (Fig. 5c). **d** Heatmap showing fold-change of induced or repressed genes upon conditioning with NT or HAS2KO fSFs. Shown are all genes significantly induced or repressed in CD4+ T cells by both NT and HAS2KO fSFs. Red corresponds to upregulated genes, blue corresponds to downregulated genes. **e** Heatmap showing fold-change of conditioned DEGs unique to HAS2KO fSF conditioning (not present upon NT fSF conditioning) as compared to UT CD4+ T cells. Red corresponds to upregulated genes, blue corresponds to downregulated genes.

To better understand the impact of fibroblasts on CD4+ T cells, we next conducted RNA sequencing (RNAseq) experiments. CD4+ T cells from four independent donors were cultured for 24 h with either media alone (UT) or with NT or HAS2KO fSFs. Although fSFs are adherent cells, there was a possibility that some would carry over upon collection of the co-cultured CD4+ T cells, which could markedly affect the RNAseq results. To account for this, we also carried out RNAseq on purified fSFs; this allowed us to identify and separate out the repertoire of fSF-specific transcripts. Principal component analysis (PCA) (Fig. 5a) showed that relative to CD4+ T cells, purified fSFs occupied a distinct region of PCA space, as expected. Non-conditioned CD4+ T cells were also well separated from CD4+ T cells cultured with NT or HAS2KO fSFs, suggesting a potent transcriptional response of CD4+ T cells upon direct contact with the fibroblasts. Interestingly, PCA of just the conditioned CD4+ T cells demonstrated a clear segregation of the T cells conditioned by NT vs. HAS2KO fSFs (Fig. 5b), suggesting subtle but noticeable differences in the effect of HA-sufficient vs. HA-deficient fibroblasts.

To identify genes modulated by fibroblast conditioning, we first compiled the differentially expressed genes (DEGs) between conditioned and non-conditioned CD4+ T cells, and then subtracted the genes specific to fibroblasts, identified as DEGs between fibroblasts and T cells (Fig. 5c). This resulted in a list of 61 DEGs in CD4+ T cells conditioned by NT fSFs, and 50 DEGs in CD4+ T cells conditioned by HAS2KO fSFs. An overview of these genes by hierarchical clustering is presented in Fig S3a. Hereafter, we refer to the DEGs between conditioned and non-conditioned CD4+ T cells, subtracted of putative fibroblast genes, as simply “conditioned DEGs”.

### HAS2KO fSFs upregulate genes associated with HIV permissivity and downregulate genes involved in cellular immune defense and programmed cell death

To first establish an overview of the CD4+ T cell genes whose expression is generally affected by fSF conditioning, we evaluated the overlapping conditioned DEGs from the NT and HAS2KO datasets. Among the 61 DEGs conditioned by NT fSFs and 50 DEGs conditioned by HAS2KO fSFs were 23 overlapping genes (Fig. 5d). Upregulated genes included suppressor of cytokine signaling 1 (*SOCS1*), a host factor that has been found to be important for intracellular Gag-trafficking,^13,14^ as well as IL15RA, a receptor important for IL15-driven homeostatic proliferation. These factors may facilitate completion of the HIV replication cycle whilst promoting survival and expansion of HIV-infected cells. Downregulated genes included regulators of the superfamily of GTPases Ras and Rab interactor 3 (*RIN3*),^15^ *BBC3* (also known as *PUMA*),^16,17^, and necrosis-inducing protein AlkB homolog 7 (*ALKBH7*).^18,19^ As all these genes have key roles in cellular immune defense pathways, and *BBC3* and *ALKBH7* promote cell death, downregulation of these genes may render CD4+ T cells more prone to surviving infection by HIV.

To assess what underlies the superior ability of HAS2KO fSFs to reprogram CD4+ T cells into an elevated state of HIV permissivity, we then examined which conditioned DEGs were different between the NT and HAS2KO fSF treatment conditions (Fig. 5e). Among the upregulated conditioned DEGs exclusive to the HAS2KO fSF treatment, the biggest fold change was observed in the immunoglobulin superfamily member *ICAM-1* (CD54), a synapse protein associated with increased cell-to-cell transmission of HIV,^20–23^ which was induced ~10-fold upon HAS2KO conditioning (Fig. 5e). Other upregulated genes unique to the HAS2KO set included metallothionein-1E (*MT1E*) metal-binding protein, an important mediator of immunity and anti-inflammatory responses as well as leukocyte chemotaxis and T-cell activation,^24–27^ and *BCL6*, the lineage-defining transcription factor of T follicular helper (Tfh) cells (Fig. 5e). As Tfh cells are known to be permissive to HIV and express diminished levels of multiple viral restriction factors,^28^ we then focused on a series of HIV-related restriction factors and found that both *GBP5* and *SAMHD1* were downregulated upon NT and HAS2KO fSF conditioning (Fig S3b). Among genes downregulated by HAS2KO fSFs but not NT fSFs was a member of the membrane-associated RING-CH-type finger (MARCH) family of E3 ubiquitin ligases, *MARCH8*, which encodes a protein with multiple roles in immune regulation.^29^ Overall, the RNAseq analyses suggest that fibroblasts condition CD4+ T cells by inducing a state of increased permissivity characterized by diminished innate immune recognition and enhanced cell survival, which is further accentuated when HA is diminished on the fibroblasts.

### Fibroblasts from male and female mucosa condition CD4+ T cells through similar cellular pathways

**Fig. 6:**
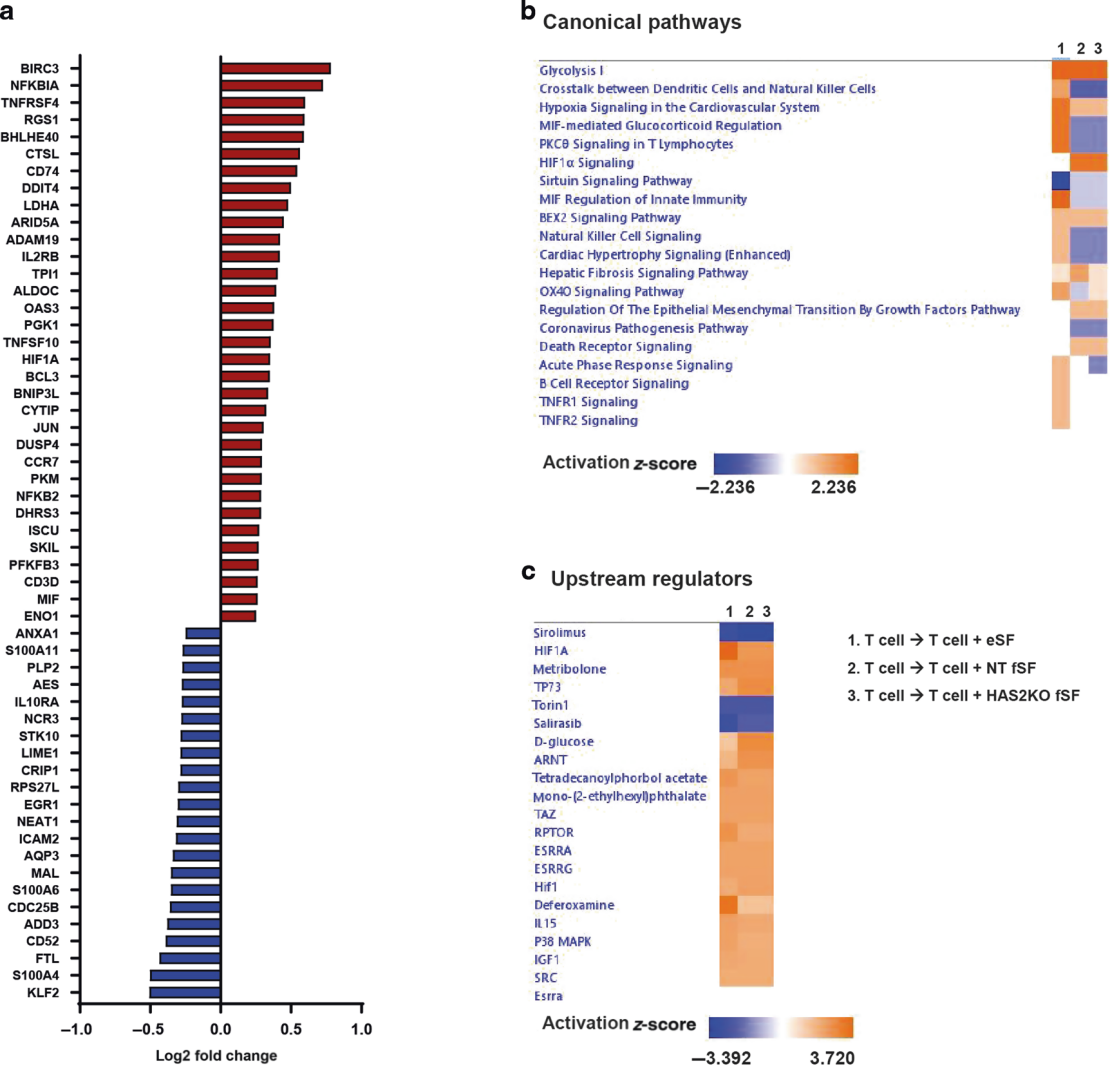
Fibroblasts regulate expression of several canonical signaling pathways in CD4+ T cells. **a** Heatmap showing log2 FC of genes differentially expressed in eSF-conditioned CD4+ T cells relative to UT CD4+ T cells as determined by scRNAseq. Red corresponds to upregulated genes, blue corresponds to downregulated genes. **b** Heatmap showing activation z-scores of the top 15 canonical pathways regulated in CD4+ T cells conditioned by eSFs (1), NT fSFs (2) or HAS2KO fSFs (3). **c** Heatmap showing activation z-scores of upstream regulators of canonical pathways induced in CD4+ T cells conditioned by eSFs, NT fSFs, and HAS2KO fSFs.

Finally, as fibroblasts from the female reproductive tract also enhance HIV infection of CD4+ T cells,^2^ we assessed whether these cells signal to CD4+ T cells in a manner similar to that mediated by fSFs. Endometrial stromal fibroblasts (eSFs) were cultured with purified activated CD4+ T cells from two independent donors for 24 h. A fraction of the T cells were then exposed to HIV to assess susceptibility to infection, while the remainder was processed for RNAseq analysis. Consistent with previous data,^2^ eSF-conditioned CD4+ T cells were more susceptible to HIV infection (Fig S4). To avoid issues of fibroblast contamination, analysis of the conditioned CD4+ T cells was conducted by single-cell RNAseq, which enabled us to gate out contaminating fibroblasts. Conditioned CD4+ T cells differentially expressed a collection of genes relative to their non-conditioned counterparts (Fig. 6a). Among the top genes upregulated by eSF-conditioning was *BIRC3*, also known as *cIAP2*.^30^ *cIAP2* inhibits tumor necrosis factor alpha (TNFα)-induced death^30,31^ and is an important regulator of nuclear factor-kappaB (NF-кB) activation.^32^ This was further supported by *NFKBIA* also being highly induced. *TNFRSF4*, encoding the T cell activation marker OX40, which is preferentially expressed on HIV-susceptible cells,^33^ was also highly upregulated by eSFs.

In order to conduct an unbiased analysis of conditioning-associated DEGs common between the two types of fibroblasts, we next implemented ingenuity pathway analysis (IPA). Although overall regulation of canonical pathways differed between CD4+ T cells conditioned by eSFs vs. fSFs, the pathway “Glycolysis I” was upregulated following conditioning by both types of fibroblasts (Fig. 6b). Glycolysis is one of the key components of cell metabolism^34,35^ and known to be a major determinant of HIV susceptibility.^36^ Remarkably, the upstream regulators of these canonical pathways (Fig. 6c) were similar between cells conditioned by eSFs vs. fSFs. For example, the mammalian target of rapamycin (mTOR)-inhibitors sirolimus and torin1 as well as salirasib (a Ras inhibitor) were all significantly downregulated in both eSF- and fSF-conditioned T cells, while the upstream regulator *HIF1A*, part of the hypoxia-responding transcription factor HIF1,^37^ was upregulated. Collectively, these data suggest that fibroblasts from both male and female HIV transmission sites condition CD4+ T cells in ways that affect their metabolic state resulting in their increased permissivity to HIV infection.

## Discussion

In this study, we demonstrate that cell-associated HA negatively regulates the ability of mucosal fibroblasts to enhance HIV infection of CD4+ T cells. We find that diminished HA production by the fibroblasts increases their ability to directly transfer infectious HIV virions to CD4+ T cells as well as to make CD4+ T cells more permissive to HIV infection. Increased permissiveness, observed with fibroblasts from both the male and female reproductive tracts, is associated with downregulation of innate immune signaling and cell death pathways, and an alteration in the metabolic state of the cells.

Prior work from our group demonstrated that one mechanism by which mucosal stromal fibroblasts enhance HIV infection of CD4+ T cells was through *trans*-infection,^2^ as defined by the ability of HIV-pulsed fibroblasts to transfer infectious virus to CD4+ T cells. A more recent study demonstrated that FRCs in secondary lymphoid organs also enhance HIV infection of cellular targets efficiently via *trans*-infection, following binding of HIV to HA on the FRCs.^11^ Our current data suggest a different role for HA on mucosal fibroblasts. We found that while diminished HA levels on fibroblasts did not affect the ability of the fSFs to capture HIV, they *increased* the fSFs’ ability to *trans*-infect CD4+ T cells. We postulate that in mucosal fibroblasts, diminished cell-associated HA levels improve cellular contact between the fibroblasts and CD4+ T cells, thereby increasing the efficiency of *trans*-infection. Future work will be needed to identify the receptor–ligand interaction that enables mucosal fibroblasts to efficiently *trans*-infect CD4+ T cells.

In comparison to NT fSFs, HAS2KO fSFs supported *trans*-infection more efficiently and better conditioned CD4+ T cells for HIV infection. As the nature of CD4+ T-cell conditioning by fibroblasts has not previously been examined, we characterized effects on the CD4+ T-cell transcriptome by RNAseq for both HA-sufficient and HA-deficient fibroblasts. fSF-conditioned CD4+ T cells markedly downregulated the pro-apoptotic gene *BBC3/PUMA* and upregulated the anti-apoptotic *BIRC3*/*cIAP*, suggesting that mucosal fibroblasts may promote HIV infection in part by promoting the survival of infected cells. Consistent with this, IPA also revealed downregulation of two inhibitors of the mTOR signaling pathway, which is important for cell proliferation and survival.^38^ Interestingly, HIV itself also promotes survival of the cells they infect by upregulating BIRC5, which acts through the OX40 signaling pathway.^39,40^ As we found that OX40 was upregulated by CD4+ T cells upon fibroblast conditioning, it may be that fibroblasts and HIV signal through similar anti-apoptotic pathways, ultimately leading to increased survival of infected cells.

Importantly, HAS2KO fSFs also uniquely regulated expression of multiple genes in CD4+ T cells. The gene most highly induced by HAS2KO fSFs conditioning was *ICAM-1*. ICAM-1 levels have previously been associated with increased productive infection of CD4+ T cells,^20^ and with inflammatory responses and HIV disease progression.^41^ Furthermore, upregulation of ICAM-1 promotes HIV-mediated syncytia formation and viral spread.^21–23^ These mechanisms, alone or in combination, could explain the increased ability of HA-deficient fibroblasts to augment HIV infection of T cells. On the other hand, conditioning by HAS2KO fSFs downregulated the Membrane-associated RING-CH 8 (*MARCH8)* gene. MARCH8 is known to remove HIV-1 Envelope glycoproteins (Env) from the cell surface, leading to new virions with limited Env expression and poorer infectivity.^42–44^ Whether downregulation of MARCH8 in CD4+ T cells by HAS2KO fibroblasts promotes their ability to propagate infection remains to be tested.

**Fig. 7:**
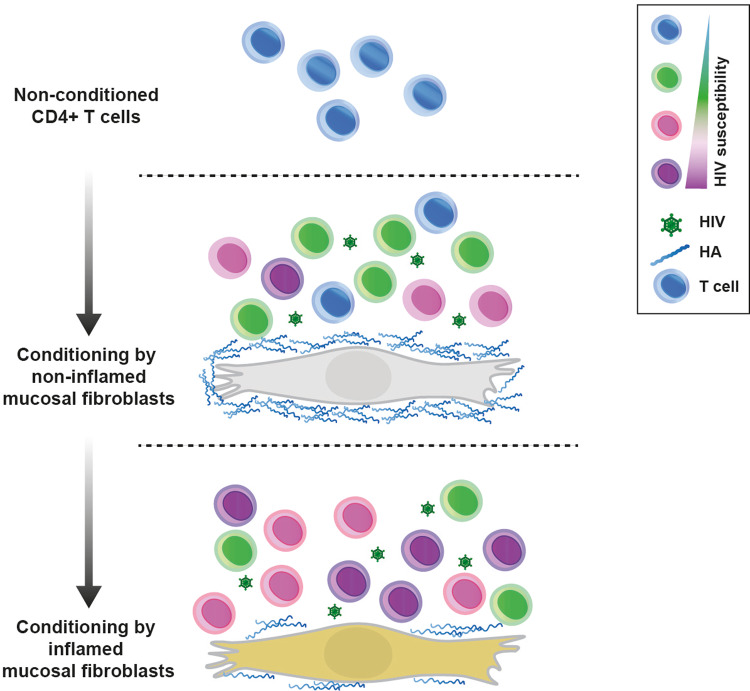
Model of the mechanism by which fibroblast conditioning enhances HIV infection of CD4+ T cells. Unconditioned CD4+ T cells that have not recently encountered mucosal fibroblasts exhibit relatively low susceptibility to HIV infection. When these cells come into direct contact with fibroblasts, they are conditioned into an elevated state of permissivity. Compared to inflamed fibroblasts, non-inflamed fibroblasts have a thick shell of high molecular weight HA surrounding the cell membrane, which somewhat dampens their ability to condition CD4+ T cells. Not shown in the model is the ability of HA-deficient fibroblasts to also better *trans*-infect CD4+ T cells.

As degradation of HA is associated with an increased inflammatory state, our HAS2KO co-culture model mimics a state of increased genital inflammation, which is known to increase HIV transmission risk.^45–47^ One cause of genital inflammation is co-infection with sexually transmitted microbial pathogens, which increases the risk of HIV acquisition.^48–53^ In particular, ulcerative STIs such as herpes simplex virus type 1 and 2 (HSV-1 and -2) are common in HIV-infected individuals, and may increase the risk of HIV acquisition in uninfected individuals.^54–56^ A recent meta-analysis found that the adjusted risk of HIV acquisition following HSV-2 infection was almost five times the risk without infection, and this was attributed to HSV-2-induced abrasions in the protective epithelial layer and recruitment of activated CD4+ T cells.^57^ Genital inflammation induced by other means can also increase HIV transmission risk, as exemplified by the failed vaginal microbicide candidate nonoxynol-9 (N9), which increased rather than decreased HIV infection rates due to its inadvertent ability to increase genital inflammation.^58^ Of note, the genital microbiome of both men and women have also been associated with inflammation and HIV transmission risk.^59,60^ We propose that under conditions of elevated genital inflammation, multiple mechanisms increase risk of HIV transmission. Activated and permissive CD4+ T cells are recruited to the genital tract, and by coming into contact with mucosal fibroblasts become more efficiently infected by HIV. Due to the inflammatory environment, these fibroblasts will have low levels of cell-surface HA, further increasing their ability to efficiently promote infection of the infiltrating CD4+ T cells (Fig. 7). Improving our understanding of this inflammation-dependent infection-promoting activity of fibroblasts may help in designing more effective strategies against HIV transmission.

## Methods

### Isolation of human primary foreskin cells

A de-identified foreskin biopsy was obtained from a healthy adult undergoing circumcision due to phimosis at Aarhus University Hospital. The patient provided informed consent prior to donation of tissue and the project was approved by the National Committee on Health Research Ethics (Study Approval #1708178). Primary foreskin fibroblasts were isolated using methods similar to those previously described.^61^ Briefly, immediately after biopsy collection, the foreskin tissue was put into a sterile 50-ml Falcon tube filled with PBS without Ca^2+^Mg^2+^ (Biowest, #L0615) containing 1% penicillin/streptomycin (p/s) (Gibco, #15140-122) and placed on ice for 30 min. The tissue was then placed in a 100-mm Petri dish and rinsed with 75% ethanol for 5 min followed by 3 washes in PBS in a new Petri dish. Any subcutaneous fat or capillaries were carefully removed and the tissue was cut into 5 × 5-mm^2^ pieces. To dissociate the epidermis from the dermis, the tissue pieces were incubated overnight in a clean Petri dish containing RPMI medium (Sigma, #R8758) with 10% FCS (Sigma, #F7524), 1% l-glutamine (Gibco, #25030-024), and 1% p/s (termed RPMI10) media supplemented with 2.4 U/ml Dispase II (Sigma-Aldrich, #D4693). The following day, the tissue was washed in PBS to remove excess Dispase before the epidermis was separated from the dermis using forceps. The dermal layer pieces were then incubated in RPMI10 supplemented with 2 mg/ml collagenase type IV-S (Sigma-Aldrich, #C1889) and 200 U/ml DNase I (Sigma-Aldrich, #10104159001) for 3–6 h with occasional mixing. The digestion was then inactivated with 5 ml FCS and the tissue was disrupted by vortexing for 30 s. The cell suspension was filtered through a 70-µm nylon cell strainer (Corning, #431751) and centrifuged at 200 × *g* for 5 min at room temperature. The supernatant was discarded and the cells were washed before they were cultured in DMEM medium (Sigma, #D6429) with 10% FCS, 1% l-glutamine, and 1% p/s (termed DMEM10 media).

### Design of single-guide RNAs (sgRNAs)

sgRNAs targeting HAS1, HAS2, and HAS3 were designed using the Synthego and CRISPOR (http://crispor.tefor.net/) web tools. Two sgRNAs were designed for each target and were synthesized by Synthego with three terminal nucleotides at both ends chemically modified with 2′-O-methyl-3′phosphorothiate. The sgRNA target sequences are shown in Table S1.

### Generation of HAS knockout fibroblasts

A third-generation lentiviral (LV) transfer plasmid backbone (LentiCRISPRV2, Addgene 52961), containing Cas9 and a sgRNA scaffold, was used for cloning of sgRNAs targeting HAS1, HAS2, and HAS3. The transfer plasmid was kindly provided by Emil Aagaard Thomsen (Giehm Lab, Aarhus University) and used to generate individual KO cell lines of HAS1, HAS2, and HAS3 in primary foreskin fibroblasts. A NT control sgRNA from GeckoV2 Human library part A with no identified target in the human genome was used as a control for unspecific Cas9 cutting. For LV production, HEK293T cells were co-transfected with the transfer plasmid as well as the packaging plasmids pRSV-REV, pMD2.G, and pMDLg/p-RRE, using PeiMax as transfection agent. Supernatants were harvested and filtered, and LV particles were concentrated by ultracentrifugation. Primary foreskin fibroblasts were transduced with the LV particles under puromycin selection to generate the three individual HAS KO lines, as well as the NT control. Two sgRNAs were designed for each HAS enzyme, and the guide generating the highest INDEL score for each target was selected for further confirmation of functional KO using an HA ELISA.

### Validation of HAS knockout in fibroblasts

To assess KO efficiency, 10^5^ cells of each HAS KO and control fibroblast cell line were lysed in 100 µl QuickExtract buffer and genomic DNA extracted, according to the manufacturer’s instructions (QuickExtract™ DNA Extraction Solution, Lucigen, #QE09050). Forward and reverse primers spanning 300–400 nucleotides up- and downstream of each target site were designed using the NCBI Primer-BLAST tool and synthesized by TAG Copenhagen A/S (Table S2). PCR reactions were run according to the manufacturer’s instructions (Phusion Polymerase Mix, Thermo Scientific, #F531S). Amplified genomic DNA fragments were subsequently purified and Sanger-sequenced (Eurofins Genomics). Efficient KO was confirmed by the Synthego ICE software (https://ice.synthego.com/#/). To validate functional KO by ELISA, fibroblasts were seeded in DMEM10. The day before supernatant harvest, medium was changed to DMEM media lacking FCS (DMEM0), as serum contains high amounts of HA. Supernatant was harvested 16 h after incubation and analyzed using the Hyaluronan Quantikine ELISA kit (R&D, #DHYAL0), following the manufacturer’s instructions.

### PBMC purification and T cell isolation

PBMCs were obtained from the Stanford Blood Bank in California or from the Blood Bank at Aarhus University Hospital in Denmark. PBMCs were purified from buffy coats by Ficoll-Hypaque (GE Healthcare, #17-1440-03) density gradient centrifugation and then cryopreserved in 80% FCS, 10% RPMI10 and 10% DMSO (Sigma, #D4540). PBMCs were thawed 3 days prior to fibroblast co-culture and infection, and were activated using 1 µg/ml plate-bound anti-CD3/CD28 (#16-0037-85 and #16-0289-85, both from eBioscience) in PBS supplemented with 100 U/ml human recombinant IL-2 (PeproTech, #200-02). Where indicated, CD4+ T cells were first purified from PBMCs by immuno-magnetic negative selection with the EasySep™ Human CD4+ T Cell Isolation Kit (Stemcell, #17952).

### Fibroblast and CD4 T cell co-culture

Fibroblasts were seeded in 96-well flat-bottom cell culture plates (Nunc, #167008) and cultured in DMEM10 for 2–3 days until confluent. PBMCs or purified CD4+ T cells, activated as described above, were added to the fibroblasts at a ratio of 10:1 (10^5^ PBMCs/CD4+ T cells added to 10^4^ fibroblasts) or to wells with media alone. A lab-adapted R5-tropic GFP-reporter HIV virus strain, HIV^GFP^ (HIV BaL^2^), was added at a titrated concentration where fibroblasts most efficiently enhanced infection of PBMCs or CD4+ T cells. Cells were harvested 3 days post infection. Infection rates were determined by using an LSRII flow cytometer. Where indicated, the PBMCs were separated from the fibroblasts by a 1.0-µm pore-size transwell (Millipore, #MCRP24H48). In experiments where the effects of exogenous HA were tested, HMW HA (R&D, #GLR002) or LMW HA (R&D, #GLR001) was added to the fibroblasts (or wells lacking fibroblasts as a control) at a final amount of 10, 50 or 100 µg per 200 µl DMEM10. These concentrations of HA are within physiologically relevant ranges,^62,63^ and match those of prior studies testing effects of HA on HIV infection.^10^ Immediately after HA treatment of the wells, activated PBMCs were added to the wells and infected with HIV^GFP^ as described above. In experiments where we tested for ability of fibroblasts to *trans*-infect the CD4+ T cells, fibroblasts were pulsed with 10 pg/µl p24^Gag^ HIV^GFP^ for 2 h at 37 °C. The fibroblasts were then carefully washed three times with DMEM10 to wash away excess virus, and then co-cultured with PBMCs. Infection rates were determined 3 days post infection using an LSRII flow cytometer.

### Conditioning of CD4+ T cells

Fibroblasts were cultured in 96-well flat-bottom plates as described above. After 2–3 days, when fibroblasts had reached confluence, CD4+ T cells isolated from anti-CD3/CD28-activated PBMCs were added to the fibroblasts (either NT or HAS2KO lines), and co-cultured for 24 h. As a control, CD4+ T cells were also cultured for 24 h with only the media from the fibroblasts, and not the cells. After these 24 h of conditioning, the CD4+ T cells were separated from the fibroblasts and the co-culture media and infected with HIV^GFP^. Three days post infection, CD4+ T cells were harvested and infection rates were analyzed by an LSRII flow cytometer. See Fig. 4a for schematic illustration of setup. A similar protocol was implemented to condition CD4+ T cells with eSFs.

### Flow cytometric analysis

Cells were washed in PBS and resuspended in PBS with Zombie Fixable Viability Stain (Biolegend, #423102, #423103) as per the manufacturer’s protocol. Antibodies for surface staining were then diluted into FACS buffer (PBS + 2% FBS + 2 mM EDTA) and added to the cells. The staining panel consisted of APC/Cy7-conjugated anti-CD3 (Biolegend, #344818), PE/Cy7-conjugated anti-CD4 (Biolegend #357410) and APC-conjugated anti-CD8 (Biolegend, #344722). Stained cells were washed in FACS buffer and fixed in 1% paraformaldehyde prior to analysis. Samples were run on an LSRII cytometer (BD Biosciences). Data were analyzed using the FlowJo V10 Software (Treestar). All flow cytometric datasets were pre-gated from a lymphocyte scatter gate (FSC vs. SSC) to identify singlet cells, and then sequentially gated on live cells (Zombie negative/low) and CD3^+^CD8^−^ T cells. Productively infected cells were identified as GFP+ cells. The gating strategy is illustrated in Supplementary Fig. S2.

### HIV binding assay

Fibroblasts were seeded in a 96-well cell culture plate at a concentration of 1.5 × 10^4^ cells/well. After the cells had reached confluency, HIV^GFP^ was added at a final concentration of 1.3 pg p24^Gag^/µl. The plates were incubated for 2 h at 37 °C, after which cells were washed three times in media and then lysed with the lysis buffer from the Takara p24 ELISA kit. HIV^GFP^ binding to the lysed cells was quantitated by p24 ELISA as per manufacturer’s protocol (Takara, #632200).

### RNA sequencing

CD4+ T cells were purified from activated PBMCs from four independent donors. These cells were conditioned with NT or HAS2KO fSFs for 24 h, or cultured for 24 h in the absence of fibroblasts as control condition. T cells were harvested by repeated gentle pipetting under conditions that left most of the adherent fibroblasts attached to the culture well. Aliquots of the mock or fibroblast-conditioned CD4+ T cells were subjected to infection assays to confirm that conditioning led to increased infection rates. The rest of the CD4+ T cells were lysed and RNA extracted using the Roche High Pure RNA Isolation Kit (#11828665001). RNA was also isolated from NT and HAS2KO fibroblasts cultured alone as a comparison control. Quality control (QC) of the RNA samples was conducted by Novogene prior to library construction and library QC. Sequencing was performed at a depth of 30 million reads per sample with paired-end reads of 150 bp using the HISeq 4000 instrument and Illumina platform (Novogene). The output fastq files were analyzed with the CLC Genomics Workbench 12 (QIAGEN) software. Using the RNA-seq module of the workbench, all reads were trimmed and mapped to the Genome Reference Consortium Human Build 38 (GRCh38/hg38), which enabled calculations of the expression values of the total gene reads. Expression values were then normalized to CPMs (counts per million) to compare the different expression levels of specific genes resulting from the different experimental conditions. PCA plots, Venn diagrams, heatmaps, and DEG lists were generated using the designated tools in the CLC workbench. The following filters were applied: CPM > 5, absolute fold change (absFC) >1.5, and false discovery rate (FDR) *p* value of <0.05.

For generation of the scRNAseq datasets from eSF-conditioned CD4+ T cells, activated CD4+ T cells were subjected to the same conditioning protocol as with fSFs. eSFs were isolated from a de-identifed endometrial biopsy donor (IRB approval #14-15361). After conditioning, T cells were separated from the adherent eSFs through gentle pipetting, and then subjected to scRNAseq through Biorad’s ddSeq system. The Illumina/Biorad SureCell 3′ WTA kit and BioRad’s ddSeq single-cell isolator were used for the preparation of single-cell RNA libraries according to the manufacturer’s protocol. Sequencing was performed on an Illumina NextSeq 500 instrument and Illumina’s proprietary SureCell workflow was used for sequence alignment, gene counting and read deconvolution. The R package Seurat was used to limit the analysis to viable CD4+ T cells (defined as CD3+CD4+ cells), and to identify cell clusters and gene expression profiles associated with the conditioned CD4+ T cells.

### Ingenuity pathway analysis (IPA)

Lists of DEGs were generated using the CLC Genomics Workbench and imported into the IPA software (QIAGEN IPA). The Core Analysis tool was used to analyze the affected canonical pathways (up and downregulated) based on the expression ratio (log2 fold change) in the DEG lists. An FDR *p* value of 0.05 was applied as cut-off and the regulation of pathways and upstream regulators was analyzed by FDR *p* value and by calculation of a *z*-score (standard score) representing the direction of the deviation. The pathway outcomes of the DEG lists were interpreted using the Comparison Analysis tool imbedded in the IPA software.

### Statistical analyses

Statistical analyses were performed using GraphPad Prism software version 8. Unpaired Student´s two-tailed *t*-test was used for column analyses. A significant result was defined as *P* values ≤0.05. Graphs are shown as median with SEM and asterisk coding as follows: *P ≤ 0.05; **P ≤ 0.01; ***P ≤ 0.001.

## Supplementary information

Supplementary Information

## References

[1] Tebit DM, Ndembi N, Weinberg A, Quiñones-Mateu ME (2012). Mucosal transmission of human immunodeficiency virus. Curr. HIV Res..

[2] Neidleman, J. A. et al. Mucosal stromal fibroblasts markedly enhance HIV infection of CD4+ T cells. *PLoS Pathog*. **13**. 10.1371/journal.ppat.1006163 (2017). 10.1371/journal.ppat.1006163 PMC5312882 28207890

[3] Bainbridge P (2013). Wound healing and the role of fibroblasts. J. Wound Care.

[4] Kogan G, Šoltés L, Stern R, Gemeiner P (2007). Hyaluronic acid: a natural biopolymer with a broad range of biomedical and industrial applications. Biotechnol. Lett..

[5] Jiang D, Liang J, Noble PW (2011). Hyaluronan as an immune regulator in human diseases. Physiol. Rev..

[6] Jackson DG (2009). Immunological functions of hyaluronan and its receptors in the lymphatics. Immunol. Rev..

[7] Misra, S., Hascall, V. C., Markwald, R. R. & Ghatak, S. Interactions between hyaluronan and its receptors (CD44, RHAMM) regulate the activities of inflammation and cancer. *Front. Immunol*. **6**. 10.3389/fimmu.2015.00201 (2015). 10.3389/fimmu.2015.00201 PMC4422082 25999946

[8] Tammi MI, Day AJ, Turley EA (2002). Hyaluronan and homeostasis: a balancing act. J. Biol. Chem..

[9] Camenisch TD (2000). Disruption of hyaluronan synthase-2 abrogates normal cardiac morphogenesis and hyaluronan-mediated transformation of epithelium to mesenchyme. J. Clin. Investig..

[10] Li P (2014). Exogenous and endogenous hyaluronic acid reduces HIV infection of CD4+T cells. Immunol. Cell Biol..

[11] Murakami T (2018). Secondary lymphoid organ fibroblastic reticular cells mediate trans-infection of HIV-1 via CD44-hyaluronan interactions. Nat. Commun..

[12] Korin YD, Zack JA (1998). Progression to the G1b phase of the cell cycle is required for completion of human immunodeficiency virus type 1 reverse transcription in T cells. J. Virol..

[13] Ryo A (2008). SOCS1 is an inducible host factor during HIV-1 infection and regulates the intracellular trafficking and stability of HIV-1 Gag. Proc. Natl Acad. Sci. USA.

[14] Nishi M (2009). Requirement for microtubule integrity in the SOCS1-mediated intracellular dynamics of HIV-1 Gag. FEBS Lett..

[15] Prashar A, Schnettger L, Bernard EM, Gutierrez MG (2017). Rab GTPases in immunity and inflammation. Front Cell Infect. Microbiol.

[16] Nakano K, Vousden KH (2001). PUMA, a novel proapoptotic gene, is induced by p53. Mol. Cell.

[17] Han JW (2001). Expression of bbc3, a pro-apoptotic BH3-only gene, is regulated by diverse cell death and survival signals. Proc. Natl Acad. Sci. USA.

[18] Fu D, Jordan JJ, Samson LD (2013). Human ALKBH7 is required for alkylation and oxidation-induced programmed necrosis. Genes Dev..

[19] Jordan JJ (2017). ALKBH7 drives a tissue and sex-specific necrotic cell death response following alkylation-induced damage. Cell Death Dis..

[20] Tardif MR, Tremblay MJ (2003). Presence of host ICAM-1 in human immunodeficiency virus type 1 virions increases productive infection of CD4+T lymphocytes by favoring cytosolic delivery of viral material. J. Virol..

[21] Jolly C, Mitar I, Sattentau QJ (2007). Adhesion molecule interactions facilitate human immunodeficiency virus type 1-induced virological synapse formation between T cells. J. Virol..

[22] Galloway NLK (2015). Cell-to-cell transmission of HIV-1 is required to trigger pyroptotic death of lymphoid-tissue-derived CD4 T cells. Cell Rep..

[23] Fortin JF, Cantin R, Lamontagne G, Tremblay M (1997). Host-derived ICAM-1 glycoproteins incorporated on human immunodeficiency virus type 1 are biologically active and enhance viral infectivity. J. Virol..

[24] Vignesh, K. S. & Deepe, G. S. Metallothioneins: emerging modulators in immunity and infection. *Int. J. Mol. Sci*. **1****8**. 10.3390/ijms18102197 (2017). 10.3390/ijms18102197 PMC5666878 29065550

[25] Inoue, K. I., Takano, H., Shimada, A. & Satoh, M. Metallothionein as an anti-inflammatory mediator. *Mediators Inflamm*. **2009**. 10.1155/2009/101659 (2009). 10.1155/2009/101659 PMC2679981 19436762

[26] Yin, X., Knecht, D. A. & Lynes, M. A. Metallothionein mediates leukocyte chemotaxis. *BMC Immunol*. **6**. 10.1186/1471-2172-6-21 (2005). 10.1186/1471-2172-6-21 PMC1262721 16164753

[27] Rice, J. M., Zweifach, A. & Lynes, M. A. Metallothionein regulates intracellular zinc signaling during CD4+T cell activation. *BMC Immunol*. **17**. 10.1186/s12865-016-0151-2 (2016). 10.1186/s12865-016-0151-2 PMC4890327 27251638

[28] Amet T (2017). BCL6 represses antiviral resistance in follicular T helper cells. J. Leukoc. Biol..

[29] Lin H, Li S, Shu HB (2019). The membrane-associated MARCH E3 ligase family: emerging roles in immune regulation. Front. Immunol..

[30] Conte D (2006). Inhibitor of apoptosis protein cIAP2 is essential for lipopolysaccharide-induced macrophage survival. Mol. Cell Biol..

[31] Graber, T. E. & Holcik, M. Distinct roles for the cellular inhibitors of apoptosis proteins 1 and 2. *Cell Death Dis*. **2**. 10.1038/cddis.2011.20 (2011). 10.1038/cddis.2011.20 PMC3101816 21430708

[32] Mahoney DJ (2008). Both cIAP1 and cIAP2 regulate TNFα-mediated NF-κB activation. Proc. Natl Acad. Sci. USA.

[33] Cavrois M (2017). Mass cytometric analysis of HIV entry, replication, and remodeling in tissue CD4+T cells. Cell Rep..

[34] Pearce EL, Pearce EJ (2013). Metabolic pathways in immune cell activation and quiescence. Immunity.

[35] Salmond RJ (2018). mTOR regulation of glycolytic metabolism in T cells. Front Cell Dev. Biol..

[36] Sáez-Cirión A, Sereti I (2020). Immunometabolism and HIV-1 pathogenesis: food for thought. Nat. Rev. Immunol..

[37] McNamee EN, Korns Johnson D, Homann D, Clambey ET (2013). Hypoxia and hypoxia-inducible factors as regulators of T cell development, differentiation, and function. Immunol. Res..

[38] Hung, C. M., Garcia-Haro, L., Sparks, C. A. & Guertin, D. A. mTOR-dependent cell survival mechanisms. *Cold Spring Harb Perspect. Biol*. **4**. 10.1101/cshperspect.a008771 (2012). 10.1101/cshperspect.a008771 PMC3504431 23124837

[39] Kuo HH (2018). Anti-apoptotic protein BIRC5 maintains survival of HIV-1-infected CD4+T cells. Immunity.

[40] Ma T (2020). HIV efficiently infects T cells from the endometrium and remodels them to promote systemic viral spread. Elife.

[41] Yu X, Shang H, Jiang Y (2020). ICAM-1 in HIV infection and underlying mechanisms. Cytokine.

[42] Koyama T, Tada T, Fujita H, Tokunaga K (2013). Membrane-associated RING-CH (MARCH) 8 protein inhibits HIV-1 infection. Retrovirology.

[43] Tada T (2015). March8 inhibits HIV-1 infection by reducing virion incorporation of envelope glycoproteins. Nat. Med.

[44] Zhang Y (2020). MARCH8 inhibits viral infection by two different mechanisms. Elife.

[45] Passmore JAS, Jaspan HB, Masson L (2016). Genital inflammation, immune activation and risk of sexual HIV acquisition. Curr. Opin. HIV AIDS.

[46] Galvin SR, Cohen MS (2004). The role of sexually transmitted diseases in HIV transmission. Nat. Rev. Microbiol.

[47] Masson L (2015). Genital inflammation and the risk of HIV acquisition in women. Clin. Infect. Dis..

[48] Guvenc F, Kaul R, Gray-Owen SD (2020). Intimate relations: molecular and immunologic interactions between *Neisseria gonorrhoeae* and HIV-1. Front Microbiol.

[49] Machado, J. R. et al. Mucosal immunity in the female genital tract, HIV/AIDS. *Biomed. Res. Int*. **2014**. 10.1155/2014/350195 (2014). 10.1155/2014/350195 PMC4181941 25313360

[50] Mwatelah, R., McKinnon, L. R., Baxter, C., Abdool Karim, Q. & Abdool Karim, S. S. Mechanisms of sexually transmitted infection-induced inflammation in women: implications for HIV risk. *J. Int. AIDS Soc*. **22**. 10.1002/jia2.25346 (2019). 10.1002/jia2.25346 PMC6715949 31468677

[51] Cohen, M. S. Sexually transmitted diseases enhance HIV transmission: no longer a hypothesis. *Lancet.* **351**. 10.1016/s0140-6736(98)90002-2 (1998). 10.1016/s0140-6736(98)90002-2 9652712

[52] Cohen, M. S., Council, O. D. & Chen, J. S. Sexually transmitted infections and HIV in the era of antiretroviral treatment and prevention: the biologic basis for epidemiologic synergy. *J. Int. AIDS Soc*. **22**. 10.1002/jia2.25355 (2019). 10.1002/jia2.25355 PMC6715951 31468737

[53] Kalichman SC, Pellowski J, Turner C (2011). Prevalence of sexually transmitted co-infections in people living with HIV/AIDS: Systematic review with implications for using HIV treatments for prevention. Sex. Transm. Infect..

[54] Tan DHS, Kaul R, Walsmley S (2009). Left out but not forgotten: should closer attention be paid to coinfection with herpes simplex virus type 1 and HIV?. Can. J. Infect. Dis. Med. Microbiol..

[55] Des Jarlais, D. C. et al. HSV-2 co-infection as a driver of HIV transmission among heterosexual non-injecting drug users in New York City. *PLoS ONE*. **9**, e87993 (2014). 10.1371/journal.pone.0087993 PMC3909306 24498235

[56] Johnson KE (2009). Foreskin inflammation is associated with HIV and herpes simplex virus type-2 infections in Rakai, Uganda. AIDS.

[57] Looker KJ (2017). Effect of HSV-2 infection on subsequent HIV acquisition: an updated systematic review and meta-analysis. Lancet Infect. Dis..

[58] Weber J, Desai K, Darbyshire J (2005). The development of vaginal microbicides for the prevention of HIV transmission. PLoS Med..

[59] Liu CM (2017). Penile anaerobic dysbiosis as a risk factor for HIV infection. MBio.

[60] Eastment MC, McClelland RS (2018). Vaginal microbiota and susceptibility to HIV. AIDS.

[61] Rittié L, Fisher GJ (2005). Isolation and culture of skin fibroblasts. Methods Mol. Med..

[62] Cowman, M. K., Lee, H. G., Schwertfeger, K. L., McCarthy, J. B. & Turley, E. A. The content and size of hyaluronan in biological fluids and tissues. *Front. Immunol*. **6**. 10.3389/fimmu.2015.00261 (2015). 10.3389/fimmu.2015.00261 PMC4451640 26082778

[63] Tengblad, A. et al. Concentration and relative molecular mass of hyaluronate in lymph and blood. *Biochem J.* **236**, 521–525, 10.1042/bj2360521 (1986). 10.1042/bj2360521 PMC1146871 3753464

[64] Garcia JV, Miller AD (1991). Serine phosphorylation-independent downregulation of cell-surface CD4 by nef. Nature.

