# Supplementary material for: Hyaluronic acid is a negative regulator of mucosal fibroblast-mediated enhancement of HIV infection
Source: Mucosal Immunol. 2021 May 11;14(5):1203–13. doi: 10.1038/s41385-021-00409-3 (PMC8379073; doi:10.1038/s41385-021-00409-3)
Supplement: Supplementary file 1 — Supplementary Information [file 41385_2021_409_MOESM1_ESM.pdf]

SUPPLEMENTARY MATERIALS

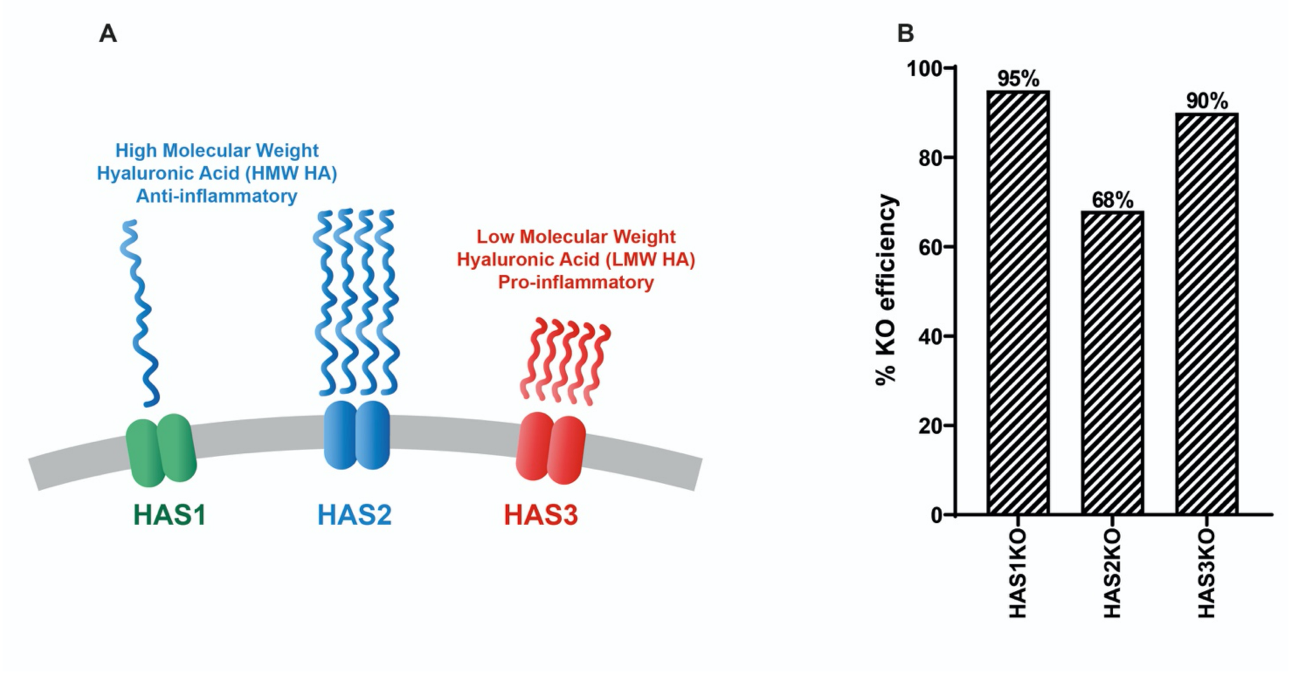

Fig S1. Generation of HAS gene knockout fibroblast lines.

A. Schematic of HA production by HAS. HA is composed of repeating disaccharides and is produced by mucosal fibroblasts through the activity of three synthases: HAS1, HAS2 and HAS3. HAS2 is the primary producer of HA and generates high molecular weight (HMW) HA. HMW HA is also produced in small amounts by HAS1. Low molecular weight (LMW) HA is mainly produced by HAS3, as well as through the enzymatic degradation of HMW HA (not shown).

B. Efficiency of HAS1, HAS2 and HAS3 gene knockout in primary foreskin fibroblasts. The fibroblasts subjected to gene disruption by CRISPR/Cas9 technology were analyzed by Synthego ICE analysis to quantitate KO efficiency. The graph shows the percentage of INDELS.

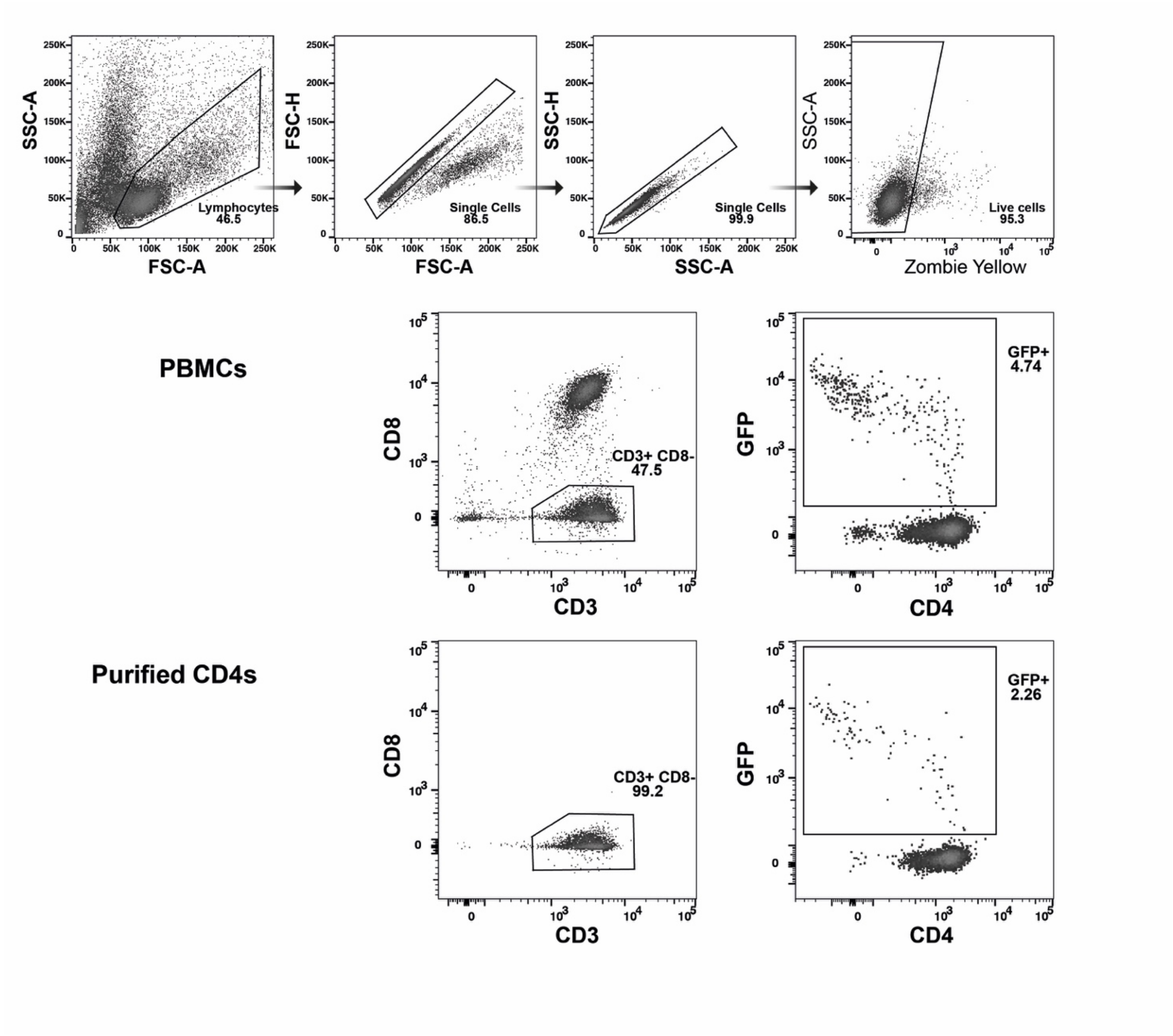

Figure S2. Flow cytometric gating strategy.

Representative dot plots showing the gating strategy to identify HIV-infected cells. Shown in the top row is the gating strategy to identify live, singlet cells. The middle row depicts gating of live, singlet cells from HIV-infected PBMC cultures for CD3+CD8- T cells, while the bottom row depicts gating of live, singlet cells from HIV-infected CD4+ T cell cultures for CD3+CD8- cells. Infected cells were identified among the CD3+CD8- population to enable identification of infected CD4+ T cells that had downregulated cell-surface CD4.

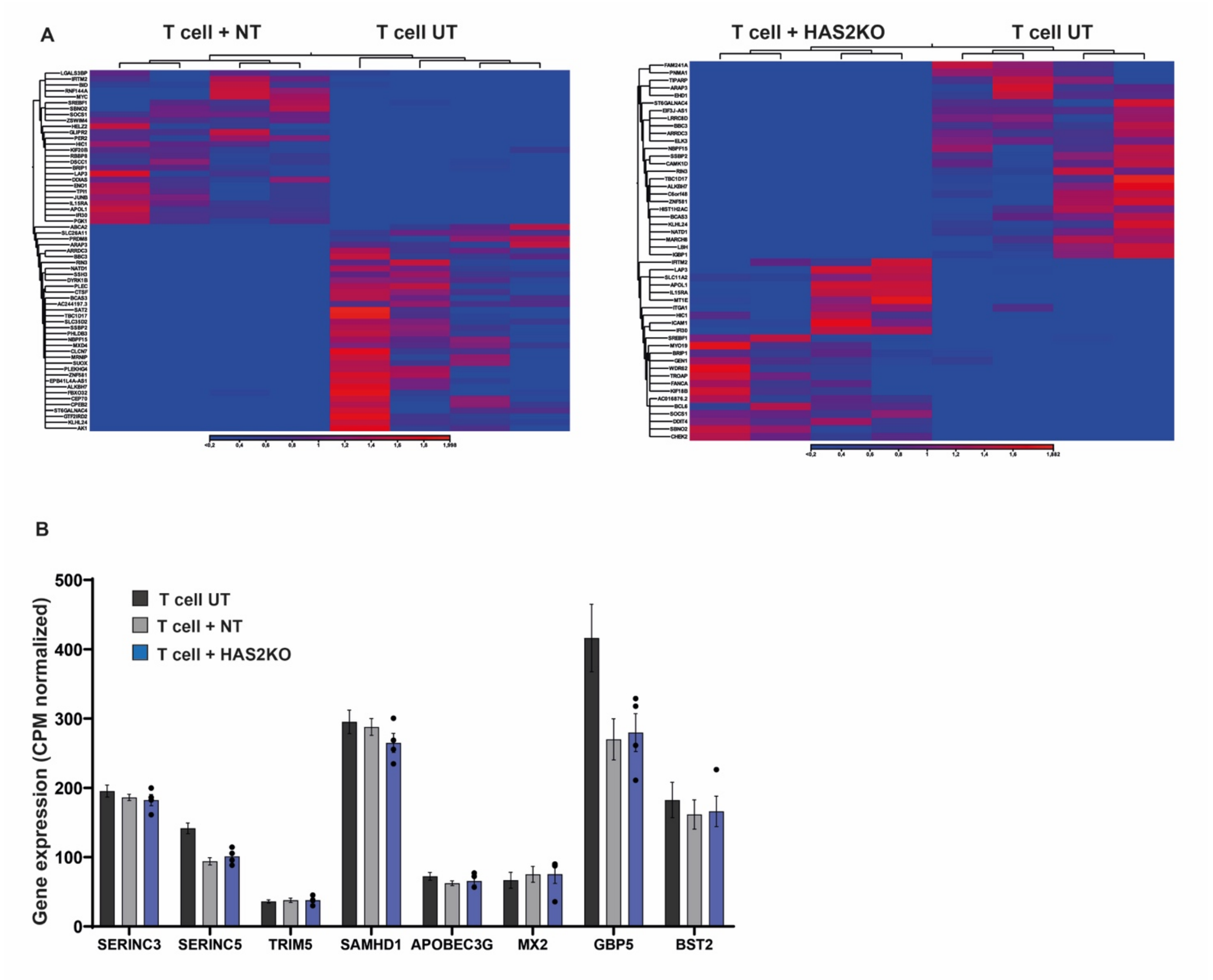

Figure S3. Differential gene expression in CD4+ T cells pre-conditioned with fibroblasts.

A. Heatmap showing absolute fold change of highlighted genes identified by Venn diagram in Fig 5b for CD4+ T cells conditioned with NT fSFs (*left*) or HAS2KO fSFs (*right*) as compared to untreated (UT) CD4+ T cells.

B. Gene expression levels of select viral restriction factors in UT CD4+ T cells or CD4+ T cells conditioned with NT or HAS2KO fSFs. Each dot represents one T cell donor.

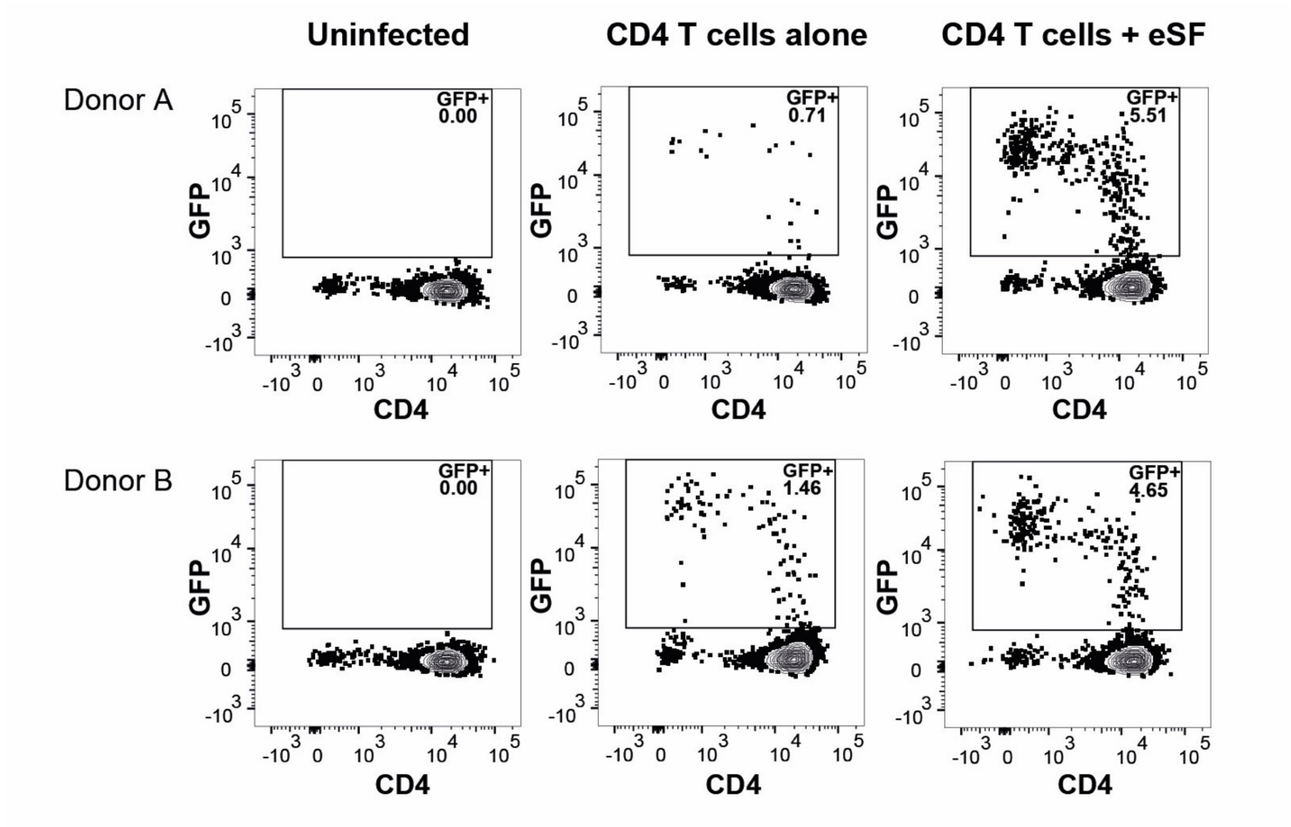

Figure S4. Confirmation of increased permissivity of eSF-conditioned CD4+ T cells to HIV infection. CD4+ T cells purified from PBMCs from two donors were activated and cultured for 24 hours with conditioned media from eSF cultures (no eSF), or directly with eSFs. The CD4+ T cells were then separated away from the conditioned media or eSFs, and exposed to the HIV BaL. Activated CD4+ T cells not co-cultured with eSFs and not exposed to HIV BaL were also included as an infection control. Viral infection levels were assessed 3 days later by flow cytometry. Cells are pre-gated on singlet, live CD3+CD8- T cells. The gates show the proportion of infected cells in each sample. Cells from the same cultures, prior to exposure to HIV BaL, were used in the scRNAseq experiments.

Name	sgRNA target sequence 5' → 3'
HAS1_sgRNA	CACCAACAGCCCCTACCCGG
HAS1_sgRNA	CGCTGATGCAGGATACACAG
HAS2_sgRNA	TGTGACTCATCTGTCTCACC
HAS2_sgRNA	TTCCACAAACTCATGCAACA
HAS3_sgRNA	GTGCATTGCCGCATACCAGG

Table S1. Sequence of single-guide RNA for CRISPR/Cas9 gene editing

Single-guide RNA (sgRNA) sequences of guides used for CRISPR/Cas9 gene editing by lentiviral delivery.

Target	Forward (Fw) vs. Reverse (Re)	Sequence
HAS1KO	Fw Primer	5'-CAGATCGGTTGCAGAGTGC-3'
HAS1KO	Re Primer	5'-CCAGTCTCATCACCAATCCC-3'
HAS2KO	Fw Primer	5'-GGGTGTTAGAAACTTCAGGGTCC-3'
HAS2KO	Re Primer	5'-CGTTACGTGTTGCGAGCTTTC-3'
HAS3KO	Fw Primer	5'-ACTGGAAATGCTGCCTCCT-3'
HAS3KO	Re Primer	5'-CACAACCCAAGGGACCTAGA-3'

Table S2. Sequence of primers to validate HAS KO.

Sequence of primers used to validate HAS knockout by Sanger sequencing. Forward and reverse primers spanning 300-400 nucleotides up- and downstream of each target site were designed using the NCBI Primer-BLAST tool (https://www.ncbi.nlm.nih.gov/tools/primer-blast/) and synthesized by TAG Copenhagen A/S.
